# Approximating the edit distance for genomes with duplicate genes under DCJ, insertion and deletion

**DOI:** 10.1186/1471-2105-13-S19-S13

**Published:** 2012-12-19

**Authors:** Mingfu Shao, Yu Lin

**Affiliations:** 1 Laboratory for Computational Biology and Bioinformatics, EPFL, Lausanne, Switzerland

## Abstract

Computing the edit distance between two genomes under certain operations is a basic problem in the study of genome evolution. The double-cut-and-join (DCJ) model has formed the basis for most algorithmic research on rearrangements over the last few years. The edit distance under the DCJ model can be easily computed for genomes without duplicate genes. In this paper, we study the edit distance for genomes with duplicate genes under a model that includes DCJ operations, insertions and deletions. We prove that computing the edit distance is equivalent to finding the optimal cycle decomposition of the corresponding adjacency graph, and give an approximation algorithm with an approximation ratio of 1.5 + *∈*.

## Introduction

The combinatorics and algorithmics of genomic rearrangements have been the subject of much research since the problem was formulated in the 1990s [1]. The advent of whole-genome sequencing has provided us with masses of data on which to study genomic rearrangements and has motivated further work. Genomic rearrangements include inversions, transpositions, block exchanges, circularizations, and linearizations, all of which act on a single chromosome, and translocations, fusions, and fissions, which act on two chromosomes. These operations are all implemented in terms of the single double-cut-and-join (DCJ) operation [2,3], which has formed the basis for much algorithmic research on rearrangements over the last few years [4-7]. A DCJ operation makes two cuts in the genome, either in the same chromosome or in two different chromosomes, producing four cut ends, then rejoins the four cut ends.

A basic problem in genome rearrangements is to compute the edit distance, i.e., the minimum number of operations needed to transform one genome into another. For unichromosomal genomes, Hannenhalli and Pevzner gave the first polynomial-time algorithm to compute the edit distance under signed inversions [8], which was later improved to linear time [9]. For multichromosomal genomes, the edit distance under the Hannenhalli-Pevzner model (signed inversions and translocations) has been studied through a series of papers [8,10-12], culminating in a fairly complex linear-time algorithm [4]; under DCJ operations, the edit distance can be computed in linear time in a simple and elegant way [2].

All of the above algorithms for computing edit distances assume equal gene content and no duplicate genes. El-Mabrouk [13] first extended the results of Hannenhalli and Pevzner to compute the edit distance for inversions and deletions. Chen *et al. *[14] studied the problem of computing the inversion distance for genomes with equal gene content in the presence of duplicate genes--a problem that comes up in determining orthologies, where greedy heuristics were used. Yancopoulos *et al. *[7] proposed some rules on how to incorporate insertions and deletions into the DCJ model, but no specific algorithms are given. Braga *et al. *[15] presented a linear-time algorithm to compute the edit distance for DCJ operations, insertions and deletions, but still without duplications. Sébastien Angibaud *et al. *[16,17] studied several model-free measures between genomes with duplicate genes; they first established a one-to-one correspondence between genes of both genomes, and then computed the measure between two genomes without duplicate genes.

In this paper, we focus on the problem of computing the edit distance between two genomes in the presence of duplications. We define the edit distance at the adjacency set level on a unit-cost model including DCJ operations, insertions and deletions (duplications are a special case of insertions). We reduce the problem of computing such an edit distance to finding the maximum number of certain cycles in the adjacency graph, Finally we give a (1.5 + *∈*)-approximation algorithm.

## Edit distance

We represent the genomes using the notations introduced by Bergeron *et al. *[2]. Denote each gene *g *with its two *extremities*, the head as *g_h _*and the tail as *g_t_*. Two consecutive genes *a *and *b *can be connected by one *adjacency*, which is represented by a pair of extremities; thus adjacencies come in four types: *a_t_b_t_*, *a_h_b_t_*, *a_t_b_h_*, and *a_h_b_h _*(there is no order for these two extremities, i.e., *a_h_b_t _*= *b_t_a_h_*). If gene *g *lies at one end of a linear chromosome, then this end can be represented by a single extremity, *g_t _*or *g_h_*, called a *telomere*. The adjacencies and telomeres of a genome form a multiset, called the *adjacency set*.

**Figure 1:**
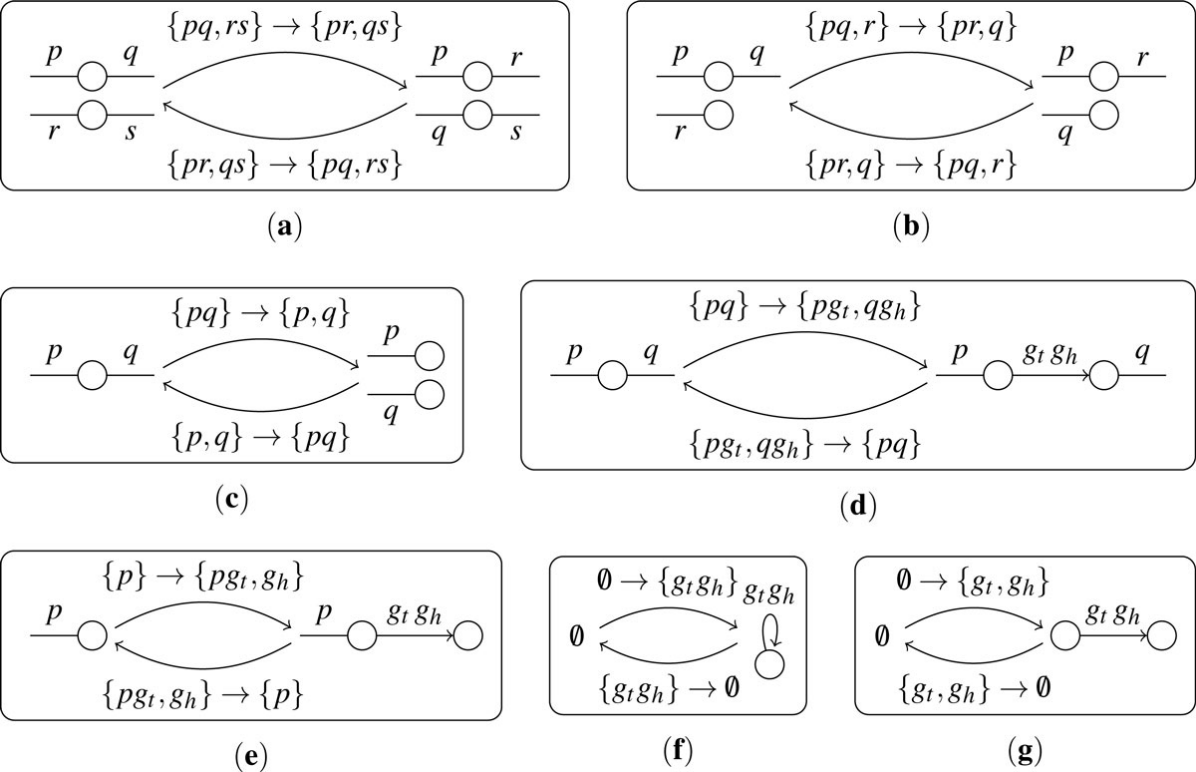
**The effect of DCJ operations, insertions and deletions on the genomic structure**. (**a**) (**b**) and (**c**) represent DCJ operations, (**d**) (**e**) (**f**) and (**g**) represent insertion and deletion. In each subfigure, the central part represents operations, and the left part and right part represent the genomic structures.

We define three operations on an adjacency set. The corresponding operations on the structure of the genome (relative positions and orientations of genes on chromosomes) are illustrated on Figure 1.

1. *DCJ (double-cut-and-join) *[2], which acts on one or two elements (adjacencies or telomeres) in one of the following ways: {*pq*, *rs*} → {*pr*, *qs*} or {*ps*, *qr*}(see Figure 1(a)); {*pq*, *r*} → {*pr*, *q*} or {*p*, *qr*}(see Figure 1(b)); {*p*, *q*} → {*pq*}or {*pq*} → {*p*, *q*}(see Figure 1(c)).

2. *Insertion*, which inserts a single gene (a pair of extremities) *g_h_g_t _*in one of the following ways: {*pq*} → {*pg_t_*, *g_h_q*} or {*pg_h_*, *g_t_q*} (see the upper arrow in Figure 1(d)); {*p*} → {*pg_t_*, *g_h_*} or {*pg_h_*, *g_t_*} (see the upper arrow in Figure 1(e)); ∅ → {*g_t_g_h_*} (see the upper arrow in Figure 1(f)); ∅ → {*g_t_*, *g_h_*} (see the upper arrow in Figure 1(g)).

3. *Deletion*, which deletes a single gene *g_h_g_t _*in one of the following ways: {*pg_t_*, *g_h_q*} → {*pq*} (see the lower arrow in Figure 1(d)); {*pg_t_*, *g_h_*} → {*p*} (see the lower arrow in Figure 1(e)); {*g_t_g_h_*} → ∅ (see the lower arrow in Figure 1(f)); {*g_t_*, *g_h_*} → ∅ (see the lower arrow in Figure 1(g)).

The *edit distance *between two adjacency sets *S*_1 _and *S*_2_, denoted as *d*(*S*_1_, *S*_2_), is the minimum number of operations (including DCJ operations, insertions and deletions) that transform *S*_1 _into *S*_2_. Here we use a unit-cost model, in which all operations have the same cost.

**Figure 2:**
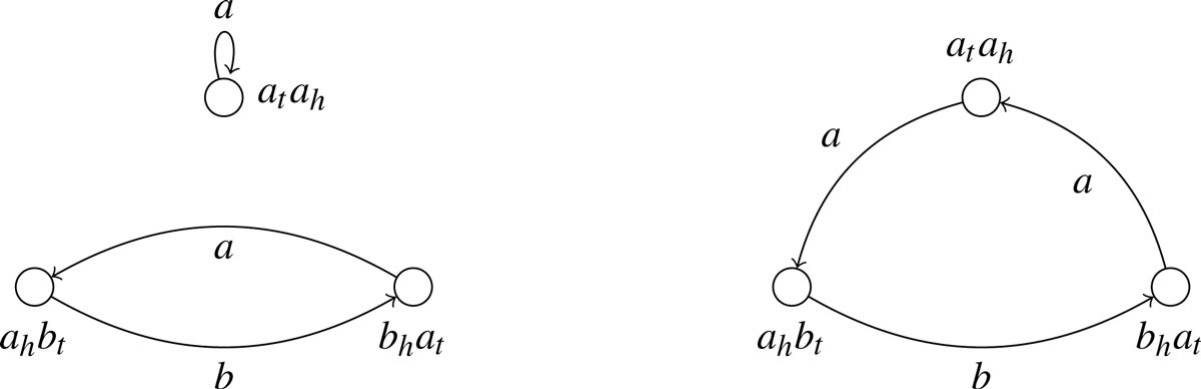
**Two genomes with different structures share the same adjacency set**. Each edge in this figure represents a gene, each node represents an adjacency.

Note that the edit distance is defined at the adjacency set level. For genomes without duplicate genes, an adjacency set denotes a unique genomic structure. However, for genomes with duplicate genes, two genomes with different structures may share the same adjacency set as illustrated in Figure 2. Thus, *d*(*S*_1_, *S*_2_) defined above is a lower bound for the edit distance between the two genomic structures. Given two adjacency sets *S*_1 _and *S*_2 _from two genomes, let *E_i _*be the multiset of extremities collected from all elements in *S_i_*, *i *= 1, 2. We pair extremities in *E*_1_\*E*_2 _into *ghost adjacencies *(named for the similar *ghost genes *of [7]) to yield the adjacency set *T*_1_; similarly, we produce *T*_2 _from *E*_2_\*E*_1_. Clearly, to transform *S*_1 _into *S*_2_, atleast |*T*_1_| deletions and |*T*_2_| insertions are needed. The following theorem shows that these insertions and deletions are both necessary and sufficient.

**Theorem 1**. *Given two adjacency sets S*_1 _*and S*_2_*, there exists an optimal series of operations with exactly |T*_1_*| deletions, exactly |T*_2_*| insertions and some DCJ operations that transforms S*_1 _*into S*_2_.

**Figure 3:**
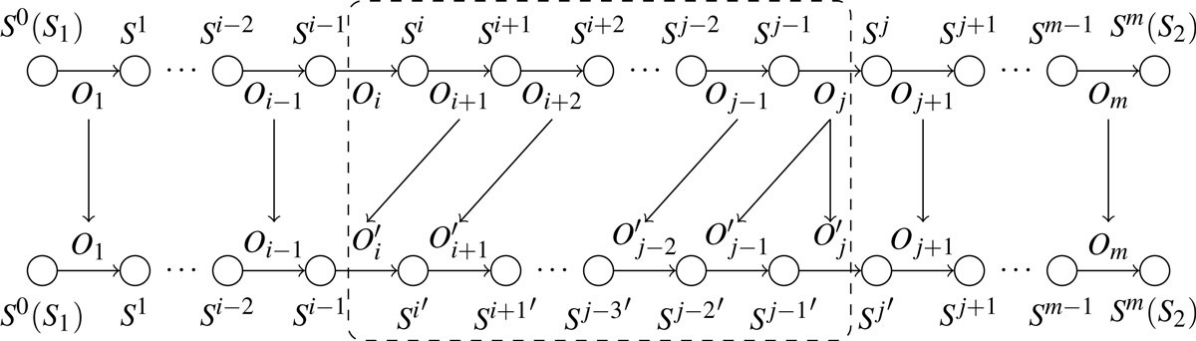
**Building a new series of operations to replace **$O_{i}O_{i+1}\cdots O_{j}$. *O_i _*will be skipped and $O_{k}^{\prime }$ will mimic *O_k _*_+ 1_for *k *= *i*, *i *+1, ..., *j *-2. Finally, $O_{j-1}^{\prime }$ and *O_j _*will be constructed according to *O_j_*.

*Proof*. We prove this theorem by contradiction. Suppose that every optimal series of operations contains more than |*T*_1_| deletions and more than |*T*_2_| insertions. Assume that *O*_1_*O*_2 _... *O_m _*is an optimal series of operations that contains a minimum number of insertions and deletions. Let *S*^0^*S*^1^*S*^2 ^... *S^m ^*be the trace of *S*_1 _in the process of transformation, where *S*^0 ^= *S*_1 _and *S^m ^*= *S*_2_. Note that for any insertion (or deletion) beyond the |*T*_1_| deletions and |*T*_2_| insertions, there must be a matching deletion (or insertion) to preserve gene content. Thus every optimal series of operations has at least a pair of insertion and deletion on the same gene. Without loss of generality, assume *O_i _*inserts a pair of extremities *g_h_g_t _*and *O_j _*deletes *g_h_g_t _*(*i *<*j*), and operations between *O_i _*and *O_j _*do not contain insertion or deletion on *g_h_g_t_*. Now we will build a new series of operations $O_{i}^{\prime }O_{i+1}^{\prime }\ldots O_{j}^{\prime }$ without the pair of insertion and deletion on *g_h_g_t _*to replace *O_i _*... *O_j_*, which produce the trace $S^{i^{\prime }}S^{i+1^{\prime }}\cdots S^{j^{\prime }}$ and satisfy $S^{j^{\prime }}=S^{j}$. This process is shown in Figure 3. Denote the two extremities inserted in *O_i _*as $g_{h}^{*}$ and $g_{t}^{*}$ to distinguish them from other *g_h _*and *g_t_*. For *k *= *i*, *...*, *j *-1, we will keep the invariant $S^{k-1^{\prime }}=\left(S^{k}\backslash \left\{p^{k}g_{h}^{*},q^{k}g_{t}^{*}\right\}\right)\cup \left\{p^{k}q^{k}\right\}$, where *p^k ^*(*q^k^*) is the extremity that shares an adjacency with $g_{h}^{*}\left(g_{t}^{*}\right)$in *S^k^*. Note that *p^k ^*or *q^k ^*might be empty if $g_{h}^{*}$or $g_{t}^{*}$ forms a telomere, or $g_{h}^{*}g_{t}^{*}$forms an adjacency in *S^k^*. Clearly this holds for *k *= *i*, since we have both $S^{i-1^{\prime }}=S^{i-1}$ and $S^{i}=\left(S^{i-1}\backslash \left\{p^{i}q^{i}\right\}\right)\cup \left\{p^{i}g_{h}^{*},q^{i}g_{t}^{*}\right\}$. To make this invariant hold for *k *= *i *+ 1, ..., *j *- 1, our new operation $O_{k-1}^{\prime }$ will mimic operation *O_k _*as follows: if *O_k _*does not affect the adjacencies or telomeres containing $g_{h}^{*}$ or $g_{t}^{*}$, then set $O_{k-1}^{\prime }=O_{k}$, and the invariant holds; if *O_k _*acts on at least one of $g_{h}^{*}$ or$g_{t}^{*}$, we will build $O_{k-1}^{\prime }$ from *O_k _*by replacing $g_{h}^{*}\left(g_{t}^{*}\right)$with *p^k ^*(*q^k^*) in *O_k_*. For example, if *O_k _*is the DCJ operation given by $\left\{p^{k-1}g_{h}^{*},cd\right\}\to \left\{p^{k-1}c,g_{h}^{*}d\right\}$, then $O_{k-1}^{\prime }$ would be {*p^k^*^-1^*q^k^*^-1^, *cd*} → {*p ^k^*^-1^*c*, *q^k^*^-1^*d*}.

Since *O_k _*does not affect, $g_{t}^{*}$ we have *q^k ^*= *q^k^*^-1^. Besides, we have *p^k ^*= *d*. Thus we have $S^{k}\backslash \left\{p^{k}g_{h}^{*},q^{k}g_{t}^{*}\right\}\cup \left\{p^{k}q^{k}\right\}=S^{k-1^{\prime }}$. Other types of operations can be expressed similarly.

Recall that *O_j _*is a deletion, i.e., {*ag_h_, bg_t_*} → {*ab*}. If *g_h _*and *g_t _*are the same as $g_{h}^{*}$ and, $g_{t}^{*}$ then we have $S^{j-2^{\prime }}=S^{j}$, and we can skip $O_{j-1}^{\prime }$ and $O_{j}^{\prime }$ in our constructed series. If *g_h _*and *g_t _*are different from $g_{h}^{*}$ and, $g_{t}^{*}$ then we have $\left\{ag_{h,}bg_{t},p^{j-1}g_{h}^{*},q^{j-1}g_{t}^{*}\right\}\subset S^{j-1}$. We can set $O_{j-1}^{\prime }$ to be {*ag_h_, bg_t_*} → {*ab, g_h_g_t_*}, and set $O_{j}^{\prime }$ to be {*p^j^*^-1^*q^j^*^-1^, *g_h_g_t_*} → {*p^j^*^-1^*g_h_, q^j^*^-1^*g_t_*}. We can verify $S^{j^{\prime }}=S^{j}$, and our constructed series contradicts the optimality of $O_{1}O_{2}\cdots O_{m}$.

## Adjacency graph decomposition

Given two adjacency sets *S*_1 _and *S*_2 _from two genomes, their corresponding *adjacency graph *is defined as a bipartite multigraph, *A *= {*S*_1 _∪ *T*_2_, *S*_2 _∪ *T*_1_, *E*},in which *u *∈ *S*_1 _∪ *T*_2 _and *v *∈ *S*_2 _∪ *T*_1 _are linked by *one *edge if *u *and *v *share one extremity, by *two *edges if they share two extremities. Note that *S*_1 _∪ *T*_2 _and *S*_2 _∪ *T*_1 _have the same set of extremities; we use *n *to denote half of the number of extremities. In the case of genomes with the same gene content and without duplicate genes, *T*_1 _= *T*_2 _= ∅, and each vertex in the adjacency graph has degree 2, which means that the adjacency graph consists of vertex-disjoint cycles and paths. We define the *length *of a cycle or a path to be the number of edges it contains. Based on Theorem 1, *T*_1 _= *T*_2 _= ∅ implies there exists an optimal solution without insertion and deletion, thus *d*(*S*_1_, *S*_2_) is just the minimum number of DCJ operations needed to transform *S*_1 _into *S*_2_. When *S*_1 _has been transformed into *S*_2_, the corresponding adjacency graph only consists of cycles of length 2 and paths of length 1. Since each DCJ operation can increase the number of cycles at most by 1, or increase the number of odd-length paths at most by 2, and we can always find out this kind of operation when *S*_1 _and *S*_2 _are different, we have *d*(*S*_1_, *S*_2_)= *n *- *c *-*o*/2, where *c *is the number of cycles and *o *is the number of odd-length paths in the adjacency graph [2].

**Figure 4:**
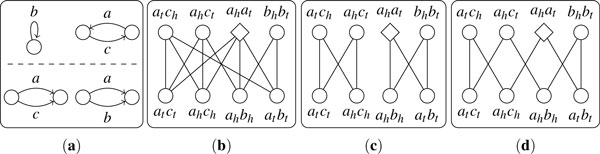
**An example of adjacency graph with duplicate genes**. (**a**) Structures of the two genomes. (**b**) Adjacency graph. (**c**) A decomposition with 2 cycles. (**d**) A decomposition with only 1 cycle. Diamonds and rectangles represent *ghost *adjacencies, and circles represent normal adjacencies.

In the presence of duplicate genes, the adjacency graph may contain vertices with degree larger than 2, so that there may be multiple ways of choosing vertex-disjoint cycles and paths that cover all vertices as illustrated in Figure 4. We say that a cycle (or path) in the adjacency graph is *alternating *if no two adjacent edges in this cycle (or path) share the same extremity. A valid *decomposition *of the adjacency graph is a set of vertex-disjoint alternating cycles and paths that cover all vertices. We say that a cycle of length ℓ is *helpful *if at most ℓ/2 - 1 vertices are *ghost *adjacencies, *unhelpful *if at least ℓ/2 vertices are *ghost *adjacencies. In fact, an *unhelpful *cycle has exactly ℓ/2 *ghost *adjacencies (all in *T*_1 _or all in *T*_2_), since adjacencies in *T*_1 _and adjacencies *T*_2 _do not have common extremities and thus cannot be linked in the adjacency graph. Now we show how to perform DCJ operations, insertions and deletions to transform *S*_1 _into *S*_2 _based on a decomposition of the corresponding adjacency graph.

**Lemma 1**. *Given two adjacency sets S*_1 _*and S*_2_*, and a decomposition D of the adjacency graph A *= {*S*_1 _∪ *T*_2_, *S*_2 _∪ *T*_1_, *E*} *with c helpful cycles and o odd-length paths, we can perform n - c - o*/2 *operations to transform S*_1 _*into S*_2_*, among which there are |T*_1_*| deletions, |T*_2_*| insertions and n - c - o*/2 *- |T*_1_*|-|T*_2_*| DCJ operations*.

*Proof*. We prove this lemma in a constructive way. We will perform operations under the guidance of the graph decomposition. The goal is to transform the adjacency graph into a collection of cycles of length 2 and paths of length 1 without ghost adjacencies, indicating that *S*_1 _has been transformed into *S*_2_. In the following, we will prove that an *unhelpful *cycle of length ℓ costs ℓ/2 operations, a path of even length ℓ costs ℓ/2 operations, a *helpful *cycle of length ℓ costs ℓ/2 -1 operations, and a path of odd length ℓ costs (ℓ - 1)/2 operations. In other words, a *helpful *cycle requires one less operation than an *unhelpful *cycle or an even-length path of the same length.

**Figure 5:**
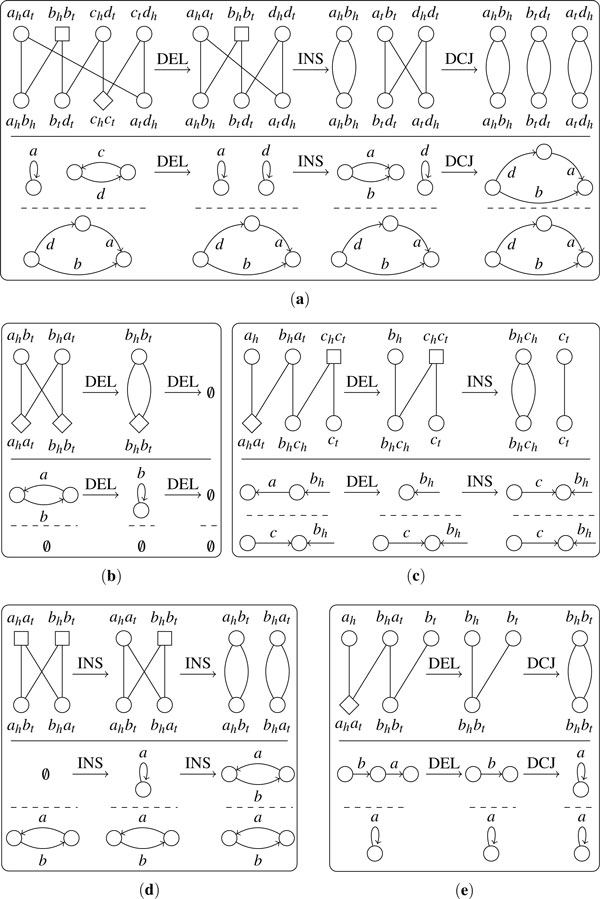
**Examples of performing operations under the guidance of decomposition**. In each subfigure, the above part shows the transformation of the adjacency graph; the below part shows the corresponding change in the genomic structure.

For a *helpful *cycle of length ℓ with *d *adjacencies in *T*_1 _and *i *adjacencies in *T*_2_, we first perform *d *deletions guided by this cycle to reduce the size of the cycle to ℓ - 2*d*. Then for each adjacency in *T*_2_, we choose one of its non-*ghost *neighbors in *S*_1 _and perform an insertion to create one more *helpful *cycle of length 2. After all adjacencies in *T*_2 _are handled, we transform the cycle of length ℓ into one of length ℓ - 2*d *- 2*i *without *ghost *adjacencies, on which finally we can perform ℓ/2 - *d *- *i *- 1 DCJ operations to create ℓ/2 - *d *- *i *cycles of length 2. An example is shown in Figure 5(a).

For a *unhelpful *cycle of length ℓ with ℓ/2 adjacencies in *T*_1_, we can perform ℓ/2 deletions to remove the adjacencies in *S*_1_. For a *unhelpful *cycle of length ℓ with ℓ/2 adjacencies in *T*_2_, we can first insert a gene as initial operand, then perform ℓ/2 - 1 insertions to create ℓ/2 cycles of length 2--see Figure 5(b)(d).

For a path with odd length ℓ, we need (ℓ - 1)/2 operations, and for a path with even length ℓ, we need ℓ/2 operations--see Figure 5(c)(e).

In sum, there are |*T*_1_| deletions, |*T*_2_| insertions and *n *- *c *- *o*/2 - |*T*_1_| - |*T*_2_| DCJ operations.

Lemma 1 states that any decomposition of the adjacency graph gives an upper bound on the edit distance. The following lemma shows that an optimal decomposition also provides a lower bound.

**Lemma 2**. $d\left(S_{1},S_{2}\right)\geq n-{\text{max}}_{D\in D}\left(c_{D}+o_{D}/2\right)$, *where * D*is the space of all decompositions of A *= {*S*_1 _∪ *T*_2_*, S*_2 _∪ *T*_1_*, E}, c_D _and o*_D _*is the number of helpful cycles and odd-length paths in D, respectively*.

*Proof*. Let ${\text{Δ}}_{P}={\text{max}}_{D\in D^{\prime \prime }}\left(c_{D}+o_{D}/2\right)-{\text{max}}_{D\in D^{\prime }}\left(c_{D}+o_{D}/2\right)$, where $D^{\prime }$ and $D^{\prime \prime }$ are the space of the decomposition before and after performing operation *P*, and *P *∈ {*DCJ, INS, DEL*}. By Theorem 1, there exists an optimal series of operations with exactly |*T*_1_| deletions and |*T*_2_| insertions. Summing over all Δ*_P _*for these operations in this optimal solution yields $\sum_{i=1}^{d\left(S_{1,}S_{2}\right)}{{\text{Δ}}_{P}}_{{}_{i}}=\left(n-|T_{1}|\right)-{\text{max}}_{D\in D}\left(c_{D}+o_{D}/2\right)$, where (*n *- |*T*_1_|) is the sum of the number of *helpful *cycles and half of the number of odd-length paths in the optimal decomposition of the adjacency graph when *S*_1 _has been transformed into *S*_2_. Define *δ_DCJ _*= 1, *δ_INS _*= 1 and *δ_DEL _*= 0. In the following, we will prove Δ*_P _*≤ *δ_P_, P *∈ {*DCJ, INS, DEL*}, which implies that $\sum_{i=1}^{d\left(S_{1,}S_{2}\right)}{{\text{Δ}}_{P}}_{{}_{i}}\leq d\left(S_{1},S_{2}\right)-|T_{1}|$. The combination of these two formulas proves this lemma.

We prove Δ*_P _*≤ *δ_P _*by contradiction. Let *A' *and *A" *be the adjacency graphs before and after performing the operation *P*. Let *σ*(*A'*) and *σ*(*A"*) be the optimal decomposition of *A' *and *A"*, respectively. Suppose Δ*_P _*>*δ_P_*, namely, (*c_σ_*(*_A"_*)+ *o_σ_*(*_A"_*)/2) - (*c_σ_*(*_A′_*;)+ *o_σ_*(*_A'_*)) >*δ_P_*. Note that *P *is reversible; we denote the reversed operation as $\hat{P}$, and $\hat{P}$ simultaneously transforms *σ*(*A"*) into a decomposition of *A'*, denoted *γ*(*A'*). Since *σ*(*A'*) is optimal, we have *c_σ_*(*_A'_*)+ *o_σ_*(*_A'_*)/2 ≥ *c_γ_*(*_A'_*)+ *o_γ_*(*_A'_*)/2. Thus, to get the contradiction, we only need to prove (*c_σ_*(*_A"_*)+ *o_σ_*(*_A"_*)/2) - (*c_γ_*(*_A'_*)+ *o_γ_*(*_A'_*)/2) ≤ *δ_P_*. Recall that *γ*(*A'*) is obtained from *σ*(*A"*) by performing operation $\hat{P}$, and both *σ*(*A"*) and *γ*(*A'*) are decompositions, which includes only vertex-disjoint cycles and paths.

If *P *is a DCJ operation, then $\hat{P}$ is still a DCJ operation. A DCJ operation may merge two cycles into one cycle, split one cycle into two cycles, merge two paths into one path, split one path into two paths, merge one path and one cycle into one path, split one path into one cycle and one path, rearrange two odd(even)-length paths into two even(odd) paths or make no change in the number of cycles and odd-length paths. Among those possible operations, the following four cases can reduce the number of *helpful *cycles or odd-length paths: (i) merge two *helpful *cycles into one *helpful *cycle; (ii) merge two odd-length paths into one even-length path; (iii) rearrange two odd-length paths into two even-length paths; (iv) merge one *helpful *cycle and one odd-length path into one odd-length path. For any of these four cases, we have (*c_σ_*(*_A"_*)+ *o_σ_*(*_A"_*)/2) - (*c_γ_*(*_A'_*)+ *o_γ_*(*_A'_*)/2) = 1. For other possible DCJ operations, we have (*c_σ_*(*_A"_*)+ *o_σ_*(*_A"_*)/2) - (*c_γ_*(*_A'_*)+ *o_γ_*(*_A'_*)/2) ≤ 0.

If *P *is an insertion, then $\hat{P}$ is a deletion. Similarly, among all possible deletions, the following five cases can reduce the number of *helpful *cycles or odd-length paths: (i) merge two *helpful *cycles into one *helpful *cycle; (ii) merge two odd-length paths into one even-length path; (iii) rearrange two odd-length paths into two even-length paths; (iv) merge one *helpful *cycle and one odd-length path into one odd-length path; (v) change a *helpful *cycle into an *unhelpful *one. For any of these five cases, we have (*c_σ_*(*_A"_*)+ *o_σ_*(*_A"_*)/2) - (*c_γ_*(*_A'_*)+ *o_γ_*(*_A'_*)/2) = 1. For other possible deletions, we have (*c_σ_*(*_A"_*)+ *o_σ_*(*_A"_*)/2) - (*c_γ_*(*_A'_*)+ *o_γ_*(*_A'_*)/2) ≤ 0.

If *P *is a deletion, then $\hat{P}$ is an insertion. A insertion may split one cycle into two cycles, split one path into two paths, or split one path into one cycle and one path. All these possible insertions will not reduce the number of *helpful *cycles or odd-length paths. Thus, any deletion will not increase the number of *helpful *cycles or the number of odd-length paths, and we have *c_σ_*(*_A"_*)+ *o_σ_*(*_A"_*)/2 ≤ *c_γ_*(*_A'_*)+ *o_γ_*(*_A'_*)/2.   □

Combining Lemma 1 and Lemma 2, we have the following theorem.

**Theorem 2**. $d\left(S_{1},S_{2}\right)=n-{\text{max}}_{D\in D}\left(c_{D}+o_{D}/2\right)$, *where  D is the space of all decompositions of A *= {*S*_1 _∪ *T*_2_, *S*_2 _∪ *T*_1_, *E*}, *c_D _and o_D _are the numbers of helpful cycles and odd-length paths in D, respectively*.

## Approximation algorithm

We design an approximation algorithm by using techniques employed on the problem of BREAKPOINT GRAPH DECOMPOSITION[5,6,18-20]. The basic idea is to find the maximum number of vertex-disjoint *helpful *cycles of length 4 in the adjacency graph. This problem can be reduced to the problem of K-SET PACKING problem with *k *= 4, for which the best-to-date algorithm has an approximation ratio of 2 + ∈ [21,22].

To make use of such algorithm, we must remove telomeres and keep only cycles in the adjacency graph. This can be done by introducing *null extremities τ *and *null adjacencies ττ*, which are different from other extremities and adjacencies (the same definition is introduced in [7]). Given two adjacency sets *S*_1 _and *S*_2 _with 2*k*_1 _and 2*k*_2 _telomeres respectively, we replace each telomere *x *by the adjacency *xτ*. If we additionally have *k*_1 _<*k*_2_, we must add (*k*_2 _- *k*_1_) null adjacencies *ττ *to *S*_1 _in order to balance the degrees. The corresponding adjacency graph is constructed in the same way as the case without null extremities: two adjacencies are linked by one edge if they share one extremity, by two edges if they share two extremities. Now we prove that this "telomere-removal" operation does not change *d*(*S*_1_, *S*_2_).

**Theorem 3**. Let *S*_1 _*and S*_2 _*be two adjacency sets and denote by *$S_{1}^{\prime }$ and $S_{2}^{\prime }$*the adjacency sets obtained from S*_1 _*and S*_2 _*by removing telomeres. Then we can write *$d\left(S_{1},S_{2}\right)=d\left(S_{1}^{\prime },S_{2}^{\prime }\right)$.

**Figure 6:**
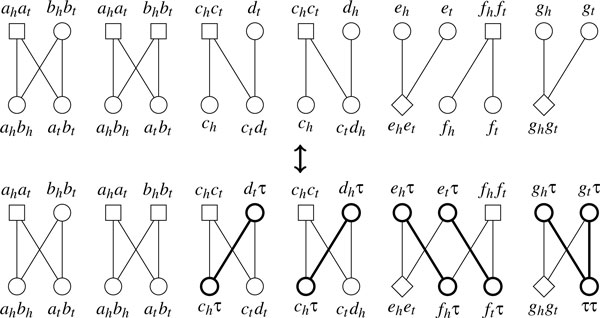
**One example of the "telomere-removal" and "telomere-recovery" process**. Thick circles represent adjacencies containing null extremities, and thick lines represent edges connecting null extremities.

**Figure 7:**
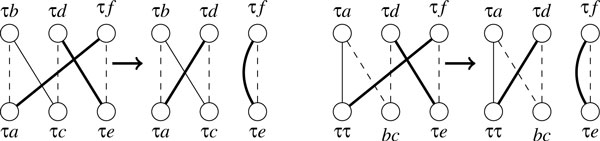
**Two cases of the adjacency graph with more than 2 edges that are linked through *τ***. Dashed lines might represent more than one edge.

*Proof*. We first prove $d\left(S_{1},S_{2}\right)\geq d\left(S_{1}^{\prime },S_{2}^{\prime }\right)$. Let *A *= {*S*_1 _∪ *T*_2_, *S*_2 _∪ *T*_1_, *E*} be the adjacency graph with respect to *S*_1 _and *S*_2 _and *σ*(*A*) be the optimal decomposition of *A*. Let $A\prime =\left\{S_{\text{1}}^{\prime }\cup T_{\text{2}},S_{2}^{\prime }\cup T_{\text{1}},E\right\}$ be the adjacency graph with respect to $S_{1}^{\prime }$ and $S_{2}^{\prime }$ and *σ*(*A'*) be the optimal decomposition of *A'*. Suppose *σ*(*A*) contains *c helpful *cycles, *o *odd-length paths and *e *even-length paths, and among these *e *even-length paths, *e*_1 _of them contain two telomeres in *S*_1 _and *e*_2 _of them contain two telomeres in *S*_2_. Suppose *S*_1 _and *S*_2 _contains 2*k*_1 _and 2*k*_2 _telomeres respectively (w.l.o.g., assume *k*_1 _≤ *k*_2_). Since an odd-length path contains one telomere in each adjacency set while an even-length path contains two telomeres in one adjacency set, we have *o *+ 2*e*_1 _= 2*k*_1 _and *o *+ 2*e*_2 _= 2*k*_2_. We can perform the following modifications on *σ*(*A*) to transform it into a decomposition of *A' *(see Figure 6). Nothing needs to be done for cycles. For odd-length paths, link their two telomeres to form a *helpful *cycle; for each even-length path with both telomeres in *S*_1_, arbitrarily choose one even-length path with both telomeres in *S*_2 _and link these two paths to form a *helpful *cycle; for the remaining *e*_2 _- *e*_1 _even-length paths, use *e*_2 _- *e*_1 _= *k*_2 _- *k*_1 _null adjacencies *ττ *to transform each such path into a *helpful *cycle. Thus, there are *c *+ *e*_2 _helpful cycles in this decomposition of *A'*, so that the upper bound on $d\left({S^{\prime }}_{1},{S^{\prime }}_{2}\right)$ is (*n *+ *k*_2_) - *c*- *e*_2 _= *n *- *c *- *o*/2 = *d*(*S*_1_, *S*_2_). Now we prove $d\left(S_{\text{1}},S_{\text{2}}\right)\leq d\left(S_{\text{1}}^{\prime },S_{\text{2}}^{\prime }\right)$. Note that *σ*(*A'*) only consists of vertex-disjoint cycles, and *unhelpful *cycles cannot contain any null extremity. We claim that, for each *helpful *cycle in *σ*(*A'*), there must be no more than two null extremities *τ *on each side. Otherwise, we can always choose two nonadjacent edges that are linked through *τ*, exchange four ends of them, and divide this cycle into two cycles (see Figure 7), contradicting the optimality of *σ*(*A'*). Now we transform *σ*(*A'*) into a decomposition of *A *by recovering all removed telomeres (see Figure 6). Each cycle falls into one of three cases: (a) it contains one *xτ *adjacency on each side, then the recovery will yield one odd-length path; (b) it contains one *ττ *adjacency on one side, then the recovery will yield one even-length path; (c) it contains two *xτ*-like adjacencies on each side, then the recovery will yield two even-length paths. In all three cases the value *n *- *c *- *o*/2 remains unchanged, and after the recovery we obtain a decomposition of *A*. Thus we have $d\left(S_{\text{1}},S_{\text{2}}\right)\leq d\left(S_{\text{1}}^{\prime },S_{\text{2}}^{\prime }\right)$.   □

In summary, based on Theorems 2 and 3, we have stated the equivalence of the problem of computing the edit distance and that of finding a valid decomposition with a maximum number of *helpful *cycles in an adjacency graph without telomeres. The latter problem is NP-hard by a reduction from the NP-hard problem--BREAKPOINT GRAPH DECOMPOSITION[23], since any instance of the BREAKPOINT GRAPH DECOMPOSITION is indeed an adjacency graph without ghost adjacencies. Thus, the problem of computing the edit distance is also NP-hard.

Now we give the approximation algorithm and prove that its approximation ratio is 1.5 + *∈*.

Approximation Algorithm

**Input: **Two adjacency sets *S*_1 _and *S*_2 _from two genomes

**Output: **A series of operations to transform *S*_1 _into *S*_2_.

**Step 1 **Add null adjacencies to *S*_1 _and *S*_2 _to obtain $S_{\text{1}}^{\prime }$ and $S_{\text{2}}^{\prime }$ without telomeres. Build the adjacency graph $A\prime =\left\{S_{\text{1}}^{\prime }\cup T_{\text{2}},S_{2}^{\prime }\cup T_{\text{1}},E\right\}$.

**Step 2 **Collect all *helpful *cycles of length 4 in *A' *as  C. Find a subset  S of  C in which no two cycles share one adjacency using the (2 + *ε*)-approximation algorithm for the K-SET PACKING problem with *k *= 4.

**Step 3 **Remove the adjacencies covered by cycles in  S. Arbitrarily decompose the remaining part of *A' *into cycles, denoting this set as $S^{\prime }$.

**Step 4 **Remove the null adjacencies of cycles in $S\cup S^{\prime }$ to obtain a decomposition of *A*. Transform *S*_1 _into *S*_2 _according to Lemma 1 guided by these cycles and paths.

The running time of the above algorithm is dominated by the time complexity of the (2 + *ε*)-approximation algorithm for the K-SET PACKING problem with *k *= 4, which is $O\left(|C{|}^{{\text{log}}_{4}1/\varepsilon }\right)$ and $|C|=O\left(n^{\text{4}}\right)$[21,22].

**Theorem 4**. *The approximation ratio of the above algorithm is *1.5 + *ε*.

*Proof*. Suppose the optimal decomposition of *A' *contain *p helpful *cycles of length 4 and *q *longer *helpful *cycles. Clearly, we have *n *≥ 2*p *+3*q*. Based on Theorem 2 and Theorem 3, we know that *d*(*S*_1_, *S*_2_) = *n *- *p *- *q*. In the algorithm, we find at least |S|*helpful *cycles, which implies that the number of operations that our algorithm outputs is at most n-|S|. Since  S is a (2 + *∈*)-approximation solution, we have (2+ε)|S|≥OPT≥p, where *OPT *is the maximum number of independent *helpful *cycles of length 4 in  C. The approximation ratio is thus

$$r\leq \frac{n-|S|}{n-p-q}\leq \frac{n-\frac{p}{2+\varepsilon }}{n-p-q}\leq 1+\frac{p+q-\frac{p}{2+\varepsilon }}{n-p-q}\leq 1+\frac{p+q-\frac{p}{2+\varepsilon }}{2p+3q-p-q}\leq 1.5+\varepsilon .$$

## Conclusion

We studied the edit distance problem for two genomes under a unit-cost model including DCJ operations, insertions (including duplications) and deletions. We proved that this problem is equivalent to finding maximum number of *helpful *cycles in the adjacency graph and gave a (1.5 + *∈*)-approximation algorithm. We made two main assumptions in this work: single-gene insertions and deletions; and unit cost for DCJ operations, insertions and deletions. Both are clearly unrealistic. For example, large segmental duplications are common in many mammalian genomes [24], paracentric rearrangements are more common than pericentric ones, at least in two Drosophila species [25], and short inversions are more common than long ones, in some prokaryotes and in the aforementioned Drosophila [26]. These constraints should be incorporated into our distance computation. Any additional constraint naturally creates complications, but we expect that at least a few natural constraints can be handled within the framework described here.

## Competing interests

The authors declare that they have no competing interests.

## Authors' contributions

MS and YL conceived the idea, performed the analysis, and wrote the manuscript. All authors read and approved the final manuscript.

## References

[B1] Fertin G Labarre A Rusu I Tannier E Vialette S Combinatorics of Genome Rearrangements 2009 MIT Press

[B2] Bergeron A Mixtacki J Stoye J A unifying view of genome rearrangements Proc 6th Workshop Algs in Bioinf (WABI'06), Volume 4175 of Lecture Notes in Comp Sci 2006 Springer Verlag, Berlin 163 173

[B3] Yancopoulos S Attie O Friedberg R Efficient sorting of genomic permutations by translocation, inversion and block interchange Bioinformatics 2005 21 16 3340 3346 10.1093/bioinformatics/bti535 15951307

[B4] Bergeron A Mixtacki J Stoye J A new linear-time algorithm to compute the genomic distance via the double cut and join distance Theor Comput Sci 2009 410 51 5300 5316 10.1016/j.tcs.2009.09.008

[B5] Chen X On sorting permutations by double-cut-and-joins Proc 16th Conf Computing and Combinatorics (COCOON'10), Volume 6196 of Lecture Notes in Comp Sci 2010 Springer Verlag, Berlin 439 448

[B6] Chen X Sun R Yu J Approximating the double-cut-and-join distance between unsigned genomes BMC Bioinformatics 2011 12 Suppl 9 S17 10.1186/1471-2105-12-S9-S17 22151948 PMC3283313

[B7] Yancopoulos S Friedberg R Sorting genomes with insertions, deletions and duplications by DCJrecombcg08 2008 170 183

[B8] Hannenhalli S Pevzner P Transforming cabbage into turnip (polynomial algorithm for sorting signed permutations by reversals) Proc 27th Ann ACM Symp Theory of Comput (STOC'95) 1995 ACM Press, New York 178 189

[B9] Bader D Moret B Yan M A fast linear-time algorithm for inversion distance with an experimental comparison J Comput Biol 2001 8 5 483 491 10.1089/106652701753216503 11694179

[B10] Jean G Nikolski M Genome rearrangements: a correct algorithm for optimal capping Inf Proc Letters 2007 104 14 20 10.1016/j.ipl.2007.04.011

[B11] Ozery-Flato M Shamir R Two notes on genome rearrangement J Bioinf Comp Bio 2003 1 71 94 10.1142/S0219720003000198 15290782

[B12] Tesler G Efficient algorithms for multichromosomal genome rearrangements J Comput Syst Sci 2002 65 3 587 609 10.1016/S0022-0000(02)00011-9

[B13] El-Mabrouk N Sorting signed permutations by reversals and insertions/deletions of contiguous segments Journal of Discrete Algorithms 2001 1 105 122

[B14] Chen X Zheng J Fu Z Nan P Zhong Y Lonardi S Jiang T Assignment of orthologous genes via genome rearrangement ACM/IEEE Trans on Comput Bio & Bioinf 2005 2 4 302 315 10.1109/TCBB.2005.48 17044168

[B15] Braga M Willing E Stoye J Genomic distance with DCJ and indels Algorithms in Bioinformatics 2010 90 101

[B16] Angibaud S Fertin G Rusu I Vialette S A pseudo-boolean framework for computing rearrangement distances between genomes with duplicatesjcb 2007 14 4 379 393 10.1089/cmb.2007.A001 17572018

[B17] Angibaud S Fertin G Rusu I Thévenin A Vialette S On the approximability of comparing genomes with duplicates Journal of Graph Algorithms and Applications 2009 13 19 53 10.7155/jgaa.00175

[B18] Caprara A Rizzi R Improved approximation for breakpoint graph decomposition and sorting by reversals J of Combin Optimization 2002 6 2 157 182 10.1023/A:1013851611274

[B19] Christie D A 3/2-approximation algorithm for sorting by reversals Proc 9th Ann ACM/SIAM Symp Discrete Algs (SODA'98) 1998 SIAM Press, Philadelphia 244 252

[B20] Lin G Jiang T A further improved approximation algorithm for breakpoint graph decomposition J of Combin Optimization 2004 8 2 183 194

[B21] Halldórsson M Approximating discrete collections via local improvements Proceedings of the sixth annual ACM-SIAM symposium on Discrete algorithms, Society for Industrial and Applied Mathematics 1995 160 169

[B22] Hurkens C Schrijver A On the size of systems of sets every t of which have an SDR, with an application to the worst-case ratio of heuristics for packing problems SIAM Journal on Discrete Mathematics 1989 2 68 72 10.1137/0402008

[B23] Kececioglu J Sankoff D Exact and approximation algorithms for sorting by reversals, with application to genome rearrangement Algorithmica 1995 13 180 210 10.1007/BF01188586

[B24] Bailey J Eichler E Primate segmental duplications: crucibles of evolution, diversity and disease Nature Reviews Genetics 2006 7 7 552 564 10.1038/nrg1895 16770338

[B25] York T Durrett R Nielsen R Dependence of paracentric inversion rate on tract length BMC Bioinformatics 2007 8 115 10.1186/1471-2105-8-115 PMC1858705 17407601

[B26] Lefebvre JF El-Mabrouk N Tillier E Sankoff D Detection and validation of single gene inversions Proc 11th Int'l Conf on Intelligent Systems for Mol Biol (ISMB'03), Volume 19 of Bioinformatics 2003 Oxford U Press i190 i196 10.1093/bioinformatics/btg1025 12855457

